# Bevacizumab for CRVO Associated CME: Effect of Timing and Frequency of Injections on Final Visual Outcome

**DOI:** 10.1155/2013/974670

**Published:** 2013-12-29

**Authors:** Joseph Pikkel, Otzem Chassid, Yumna Busool, Ward Srour, Adi Sharabi-Nov, Itzchak Beiran

**Affiliations:** ^1^Department of Ophthalmology, Ziv Medical Center, Safed, Israel; ^2^Faculty of Medicine, Bar Ilan University, Safed, Israel; ^3^Research Wing, Ziv Medical Center, Safed, Israel and Tel-Hai Academic College, Israel; ^4^Department of Ophthalmology, Rambam Medical Center, Haifa, Israel; ^5^The B. Rappaport Faculty of Medicine, Technion, Haifa, Israel

## Abstract

*Purpose*. Injection of anti-VEGF antibody into the vitreous body is a well-established treatment for ischemic central retinal vein occlusion (CRVO) associated macular edema. Various treatment regimens regarding the timing, number, and frequency of injections have been proposed. *Methods*. We reviewed the medical records of 68 patients treated by intravitreal bevacizumab (Avastin) injections for macular edema due to CRVO. We examined final visual acuity six months following the last injection in relation to injection policy (one primary injection followed by subsequent injections based on anatomical response versus a prescheduled protocol of one injection per month for the first 3 months) and in relation to the time lapsing from CRVO diagnosis to the first injection. *Results*. Mean visual acuity improved more for patients treated by a protocol of 3 prescheduled injections than for those treated with one primary injection. Improvement in mean visual acuity was greater for patients who received their first injection within the first month than those treated after 3 months (*P* < 0.01). *Conclusion*. A protocol of three prescheduled injections of bevacizumab, starting within one month of a CRVO event, was associated with better visual outcome compared to single injection and/or treatment starting more than 3 months following the time of diagnosis.

## 1. Introduction

Ischemic central retinal vein occlusion (CRVO) is a retinal vascular disorder that carries a potential risk of blindness. The introduction of anti-VEGF therapy has altered treatment options for this disease [1]. However, there is currently no standard treatment protocol for ischemic CRVO in an acute setting. Macular grid laser photocoagulation has demonstrated effectiveness in treating macular edema (ME) in branch retinal vein occlusion (BRVO) but not in CRVO [2]. Other medical and surgical therapies that have been suggested have failed to achieve the desired outcome or were associated with undesirable complications [3–10].

Vascular endothelial growth factor (VEGF), triggered by hypoxia, has been shown to increase in pathological ischemic conditions [11, 12]. In cases of CRVO, there is evidence of intraretinal upregulated expression of VEGF mRNA [13]. Increased concentration of VEGF has been reported in the vitreous fluid of patients with ischemic CRVO and plays a role in the increased vascular permeability that leads to ME [14]. Thus, the injection of anti-VEGF antibody into the vitreous body has become an accepted treatment for CRVO associated ME [15]. Following intravitreal bevacizumab injections, VEGF levels were shown to decrease considerably, to a concentration lower than physiologic levels [16].

At the present time no substantiated protocol for treating ischemic CRVO associated ME by anti-VEGF is available. Some retinal specialists inject once while others inject three times. The optimal sequence and timing of intravitreal anti-VEGF injections have yet to be determined.

In an attempt to assess the association of the number and timing of bevacizumab injections with the final visual outcome in cases of ischemic CRVO associated ME we compared retrospectively the visual outcome of patients who were treated with intravitreal anti-VEGF by different protocols in one ophthalmology department during 6-year period.

## 2. Methods

The medical records of all patients that suffered from ischemic central retinal vein occlusion and were treated with intravitreal bevacizumab (Avastin) injections for ME due to CRVO in the Ziv Medical Center, Israel, from January 1, 2006 to December 31, 2011, were reviewed. Ischemic central retinal vein occlusion was diagnosed if there was severe visual loss (equal or less than 20/200), extensive retinal hemorrhages, and cotton-wool spots, presence of relative afferent pupillary defect, poor perfusion to retina in FFA examination, and macular edema clinically and/or in OCT examination. All patients underwent an FFA examination and an OCT examination [17–19]. Data collected included age, gender, general health condition, primary visual acuity, visual acuity 6 months after last injection, timing of first intravitreal injection, and existence of macular edema 6 months after last injection. The difference between baseline and final visual acuity was calculated for each patient. Patients for whom at least 6-month follow-up data after the last injection were not available or who had incomplete medical records were excluded from the study.

Patients who were treated at the beginning of the study period (from January 2006 to June 2008) received one injection (and subsequent injections based on anatomical response). From July 2008, patients were treated according to a prescheduled protocol of one injection per month for the first 3 months.

Patients were divided into two major groups: those whose primary treatment consisted of one injection and those whose primary treatment consisted of one injection per month for 3 months. The two groups were divided into four subgroups by the timing of their first Avastin injection—within one month of the CRVO event, 1-2 months from the CRVO event, 2-3 months from the event, and 3 or more months after the CRVO event.

Statistical analyses and the Kruskal-Wallis nonparametric test were used to compare treatment outcomes among the study groups. Chi-square correlation was used to calculate the correlations between the categorical variables. *P* value <0.05 was considered statistically significant.

## 3. Results

Of 96 patients treated in our department for ischemic CRVO between January 1, 2006 and December 31, 2011, 68 met the inclusion criteria of the present study. For 29 patients, primary treatment was one intravitreal injection of bevacizumab. Of them, 6 received an additional injection and 2 received 2 additional injections. Thirty-nine were treated according to a preset protocol of three injections (one injection per month for 3 months).

**Figure 1:**
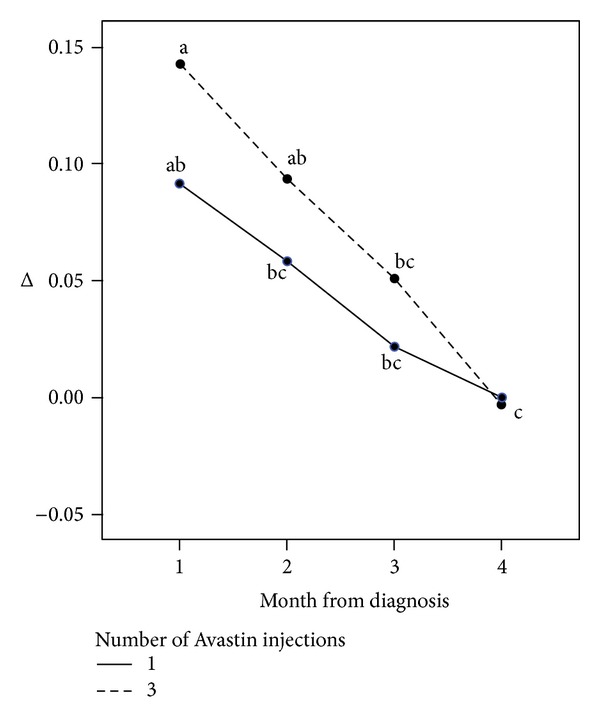
Improvement of mean visual acuity according to time of first injection and number of injections. Different subscripts represent statistically significant differences between the groups (*P* < 0.001). The 2 statistically significant results are 3 injections starting within the first month (greatest improvement) and 1 or 3 injections starting after 3 months (worst result).

Demographic data and incidence rates of selected relevant systemic diseases did not differ significantly between the two groups (Table 1).  Table 2 and Figure 1 present the improvement in visual acuity according to the time from the CRVO event to the first Avastin injection and the regimen of injections administered (1 versus 3 prescheduled injections). No improvement in visual acuity was observed among those treated according to a single or three-injection regimen. For both regimens, the earlier the administration of the first Avastin injection, the greater the improvement in visual acuity.

The number of patients treated with one injection in each subgroup and the number of patients with residual edema are shown in Table 3.

The number of patients treated with three injections in each subgroup and the number of patients with residual edema are shown in Table 4.

The improvement in mean visual acuity between those treated by 3 injections starting within the first month (best improvement) and those treated with 1 or 3 injections, starting after 3 months (worst result) was statistically significant (*P* < 0.01).

## 4. Discussion

Therapies that have been explored for treatment of CRVO, but have failed to achieve the desired outcome or were associated with undesirable complications, include laser-induced chorioretinal venous anastomosis, intravitreal administration of recombinant tissue plasminogen activator, isovolemic hemodilution therapy, oral pentoxifylline, hyperbaric oxygen therapy, radial optic neurotomy, vitrectomy with or without internal limiting membrane peeling, and direct injection of recombinant tissue plasminogen activator into the lumen of a retinal vein via retinal vein cannulation [3–10]. Intravitreal triamcinolone acetonide injections have been used as an anti-inflammatory agent to treat CRVO-ME with variable success [20]. The short acting effect and possible complications, such as increased intraocular pressure and cataract formation, led to the search of other treatment modalities for CRVO.

Until recently, most of our clinical decisions regarding the management of CRVO-ME were based on the central vein occlusion study [2]. Recently, the Cochrane Eye and Vision Group published a systematic review on anti-VEGF therapy in the management of ME secondary to CRVO [21]. They showed that ranibizumab intravitreal injections yielded good results in the short-term treatment of nonischemic CRVO-ME. Though many studies reported convincing evidence of the benefits of anti-VEGF treatments in CRVO, regarding both visual and anatomical resolution, there were no data on anti-VEGF agents among patients with ischemic CRVO, with or without ME.

The present study was conducted in order to assess injection protocols on final visual acuity in patients treated with Avastin for CRVO associated ME. Patients treated with one injection were compared to patients treated with three injections, and the effect of the timing of the first injection on treatment outcome was studied. Our results demonstrate that bevacizumab intravitreal injections applied up to 3 months following CRVO improve visual acuity. While both early first injection and multiple injections were found to have positive effect on final visual acuity, treatment timing had a greater effect than the number of injections administered. Patients who received only one early Avastin injection within 1 month of a CRVO event demonstrated greater improvement in final visual acuity than did patients who received three injections the first of which being applied more than 2 months after a CRVO event. This difference showed borderline statistical significance (*P* = 0.063). Patients who received one injection as primary treatment and had additional injections applied later showed less improvement in visual acuity than those who were treated by a preset protocol of three injections. Intravitreal injections that were administered more than three months after CRVO events were not effective in either the single or the preset three injection groups.

Due to the retrospective design of this study, reasons for the differences in timing of the first injections are unknown. Differences in patient awareness and access to medical care may be a possibility.

## 5. Conclusions 

The findings of this study demonstrate improved visual acuity following treatment of CRVO associated ME with bevacizumab intravitreal injections. The observed benefit was greater for patients for whom the first injection was administered early and for patients for whom three injections were administered (compared to a single injection). Large-scale prospective studies are needed to further substantiate and refine the results of this study.

## Figures and Tables

**Table 1:** Demographic data and incidence rates of selected diseases.

Characteristic	All patients (*n* = 68)		1 injection (*n* = 29)		3 injections (*n* = 39)		*χ* ^2^ (*P*)
	*N*	%	*N*	%	*N*	%	
Sex (number male)	42	61.8	17	58.6	25	64.1	0.212
Diagnosed with hypertension	43	63.2	17	58.6	26	66.7	0.496
Diagnosed with diabetes	27	39.7	12	41.1	15	38.5	0.808
Diagnosed with hypercoagulability	1	1.5	1	3.4	0	0	
Age: mean (range)	68.0 (29–83)		63.5 (29–79)		63.4 (49–83)		0.890
Baseline visual acuity (Mean)	6/90		6/85		6/95		0.963

**Table 2:** Mean change of visual acuity by the time from the CRVO event to the first Avastin injection and the regimen of injections administered.

Number and timing of injections	Mean primary visual acuity	Mean final visual acuity	Change in mean visual acuity
1 injection within 1 month	0.075	0.166	0.091
1 injection within 2 months	0.05	0.18	0.058
1 injection within 3 months	0.05	0.072	0.022
1 injection after more than 3 months	0.02	0.02	0.00
3 injections beginning within 1 month	0.05	0.193	0.143
3 injections beginning within 2 months	0.075	0.168	0.093
3 injections beginning within 3 months	0.04	0.091	0.051
3 injections beginning after more than 3 months	0.02	0.017	−**0.003**

**Table 3:** Patients, timing of injections, and residual edema in OCT-1 injection group.

	1 injection within 1 month	1 injection within 2 months	1 injection within 3 months	1 injection after more than 3 months
No. of patients	12	6	5	6
No. of patients with residual edema in OCT	2	1	2	4

**Table 4:** Patients, timing of injections, and residual edema in OCT-3 injections group.

	3 injection first within 1 month	3 injection first within 2 months	3 injection first within 3 months	3 injection first after more than 3 months
No. of patients	15	8	9	7
No. of patients with residual edema in OCT	2	1	2	3
